# Glycine Cleavage Powers Photoheterotrophic Growth of *Chloroflexus aurantiacus* in the Absence of H_*2*_

**DOI:** 10.3389/fmicb.2015.01467

**Published:** 2015-12-22

**Authors:** Lian He, Yaya Wang, Le You, Yadana Khin, Joseph K.-H. Tang, Yinjie J. Tang

**Affiliations:** ^1^Department of Energy, Environmental and Chemical Engineering, Washington UniversitySt. Louis, MO, USA; ^2^Department of Chemistry and Biochemistry, Clark UniversityWorcester, MA, USA; ^3^The Biodesign Institute, Arizona State UniversityTempe, AZ, USA

**Keywords:** 3-hydroxypropionate, ^13^C, C1 metabolism, CO_2_ fixation, formyltetrahydrofolate

## Abstract

*Chloroflexus aurantiacus* is an anoxygenic phototrophic bacterium. Its unique CO_2_ fixation pathway and primitive light-harvesting antenna complexes have attracted extensive research attentions. In this work, we investigated the photoheterotrophic growth of *C. aurantiacus* J-10-fl using acetate [at 55°C and without H_2(g)_]. The results indicate that glycine can promote anaerobic biomass production in a minimal medium by threefold to fivefold. Via ^13^C-metabolite analysis, we observed that glycine was involved in serine synthesis. Instead of being used as a major carbon source, glycine was degraded to produce C1 units and NAD(P)H. Tracer experiments also suggest that photoheterotrophic cultures growing with a exogenous glycine source exhibited capabilities of assimilating CO_2_ via multiple routes (including the 3-hydroxypropionate pathway). Finally, glycylglycine, a commonly used culture buffer, also significantly enhanced photoheterotrophic growth of *C. aurantiacus*, probably due to its thermal or enzymatic breakdown to glycine.

## Introduction

*Chloroflexus* *aurantiacus* is a filamentous anoxygenic phototrophic bacterium isolated from hot springs (Hanada and Pierson, 2006). It has specialized light-harvesting antenna machines and performs a cyclic photosynthetic electron transport via a type II reaction center (Tang and Blankenship, 2013). Its photosystem does not generate NADPH directly, but can convert light energy into ATP via photosynthetic electron transfer. The genome of *C. aurantiacus* strain J-10-fl has been sequenced to facilitate our understanding of its physiology and cellular metabolism (Tang et al., 2011). *C. aurantiacus* shows a versatile carbon metabolism. In aerobic and dark conditions, it can grow on various organic substrates chemoheterotrophically. *C. aurantiacus* switches to photoheterotrophic growth when supplied with acetate under anaerobic and light conditions. In the presence of H_2_/CO_2_, *C. aurantiacus* can perform a photoautotrophic growth by fixing CO_2_ via the 3-hydroxypropionate (3HOP) bi-cycle pathway (Eisenreich et al., 1993; Zarzycki et al., 2009).

In its natural habitat, *C. aurantiacus* consumes organic nutrients (e.g., short-chain fatty acids, acetate, etc.) released from cyanobacteria in the associated microbial mats (Hanada and Pierson, 2006; Lee et al., 2014). Its phototrophic metabolisms for coassimilation of organic substrates have been extensively studied. For photoheterotrophic culture of *C. aurantiacus*, the common growth medium requires complex nutrients such as yeast extract or casamino acids (Madigan et al., 1974). In some early work on photoheterotrophic cultures, both acetate and mixed gases of H_2_ and CO_2_ were provided (Strauss et al., 1992). However, we found that strain J-10-fl was unable to grow well in acetate-based minimal media with phosphate buffer but without amino acids and H_2_ supplies. To delineate key exogenous nutrients demanded by *C. aurantiacus*, we performed ^13^C-tracer experiments. The results enhanced our understanding of *C. aurantiacus* carbon and energy metabolisms, and shed lights on *C. aurantiacus* survival in the ecosystem.

## Materials and Methods

All chemicals and labeled substrates (^13^C-sodium acetate and NaH^13^CO_3,_ purity > 98%) were purchased from Sigma–Aldrich (St. Louis, MO, USA). *C. aurantiacus* strain J-10-fl was grown in a minimal PE medium at 55°C. One liter medium contained 5 mL phosphate buffer solution, 5 mL basal salt solution, 0.5 g Na_2_S_2_O_3_ and 0.5 g (NH_4_)_2_SO_4_. One liter of phosphate solution contained 75 g KH_2_PO_4_ and 78 g K_2_HPO_4_. One liter of basal salt solution contained 4.12 g Na_3_EDTA, 1.11 g FeSO_4_⋅7H_2_O, 24.65 g MaSO_4_⋅7H_2_O, 2.94 g CaCl_2_⋅2H_2_O, 23.4 g NaCl and 10 mL tracer solution. One liter of tracer solution contained 11.2 g MnSO_4_⋅4H_2_O, 2.88 g ZnSO_4_⋅7H_2_O, 2.92 g Co(NO_3_)_2_⋅4H_2_O, 2.52 g CuSO_4_⋅5H_2_O, 2.42 g Na_2_MoO_4_⋅2H_2_O, 3.1 g H_3_BO_3_ and 41.2 g Na_3_EDTA. The medium pH was adjusted to 7.5. Commercial RPMI 1640 vitamins solution (100X, Sigma–Aldrich) was added into the sterile medium. In nutrient studies, yeast extract or amino acids were added into medium to test their effects on cell growth. For photoheterotrophic cultivation, the anaerobic cultures (purged with N_2_) were grown in sealed serum bottles (containing 30 mL culture) under continuous illumination (20–30 μmol photons m^-2^ s^-1^). Neither H_2(g)_ nor CO_2(g)_ was provided in the bottle headspace. Biomass growth was monitored based on optical densities at 600 nm.

**FIGURE 1:**
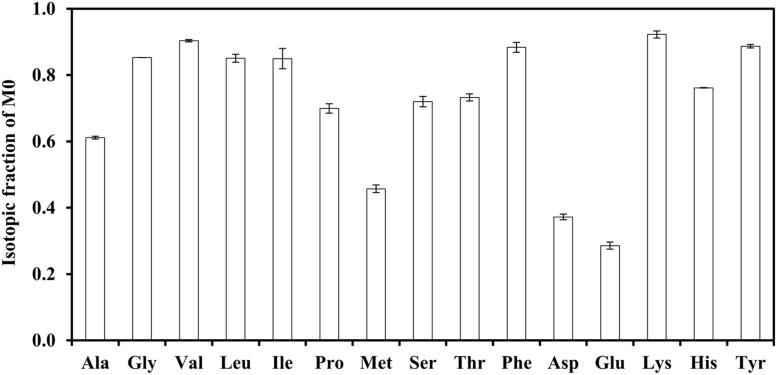
**Investigation of the key nutrients enhancing the photoheterotrophic growth of strain J-10-fl.** The medium contained 2 g/L [1-^13^C] sodium acetate and 0.2 g/L yeast extract. Unlabeled fractions (M0) of proteinogenic amino acids were mainly derived from yeast extract.

Strain J-10-fl was cultivated in four tracer media: (a) 2 g/L [1-^13^C]acetate and 0.2 g/L unlabeled yeast extract; (b) 2 g/L [1,2-^13^C_2_]acetate (fully labeled acetate) and 0.05 g/L unlabeled glycine; (c) 2 g/L [1,2-^13^C_2_]acetate, 0.05 g/L glycine, and 0.5 g/L NaH^13^CO_3_; and (d) 2 g/L unlabeled acetate, 0.05 g/L unlabeled glycine, and 0.5 g/L NaH^13^CO_3_. In each tracer experiment, exponentially growing cells from unlabeled culture were inoculated in the labeled medium at a volume ratio of 1%. The protocol for ^13^C-metabolite analysis was described elsewhere (Tang et al., 2007; You et al., 2012). In brief, the biomass was hydrolyzed in HCl solution (100°C), and the resulting amino acid mixtures were derivatized by *N*-(tert-butyldimethylsilyl)-*N*-methyltrifluoroacetamide (TBDMS). GC-MS equipped with a DB5-MS column (Agilent Technologies, USA) was used to measure amino acid labeling pattern. The mass isotopomer distributions (MIDs) of amino acids were calculated based on five MS fragments (Wahl et al., 2004): [M-159]^+^ or [M-85]^+^ (both containing the carbon skeleton of amino acids after loss of their α carboxyl groups), [M-15]^+^ or [M-57] ^+^ (both containing the whole carbon skeleton of amino acids), and f302 (containing the first and the second carbon of amino acids). The final MS results, M0, M1, and M2, represent unlabeled, singly labeled, and doubly labeled amino acids, respectively (**Figure 1**). We also measured glycine concentrations in the cultures by GC-MS. In brief, culture samples were centrifuged and supernatant was collected. After the supernatant was dried, glycine from the supernatant was derivatized by TBDMS. Meanwhile, we determined the relationship between the glycine standards and corresponding MS abundances, which were used to estimate extracellular glycine concentrations in cultures.

## Results

*Chloroflexus aurantiacus* exhibits optimal growth in photoheterotrophic conditions when the medium is supplemented with yeast extract. It can also grow well with acetate and H_2_ in a minimal medium (Strauss et al., 1992). In our experiments, strain J-10-fl supplied with yeast extract reached an OD above 1.0 within 6 days during photoheterotrophic growth. Without H_2_ and yeast extract in the minimal medium, however, photoheterotrophic cultures grew poorly. To identify the biomass building blocks that were not effectively synthesized from primary substrate acetate, photoheterotrophic cultures were supplied with ^13^C-labeled sodium acetate and unlabeled yeast extract. **Figure 1** shows the contribution of yeast extract to the synthesis of proteinogenic amino acids. Based on tracer experiments, a high fraction of ^13^C-carbon (40–70%) was incorporated into several amino acids, such as Ala, Glu, Met, and Asp, suggesting effective synthesis of those amino acids from acetate. Since the labeled carbon of [1-^13^C]acetate (purity > 98%) would be mostly incorporated into amino acids via central metabolic pathways, high unlabeled fractions of amino acids (e.g., Gly, Leu, and Phe) indicated that they were avidly absorbed from exogenous sources.

**FIGURE 2:**
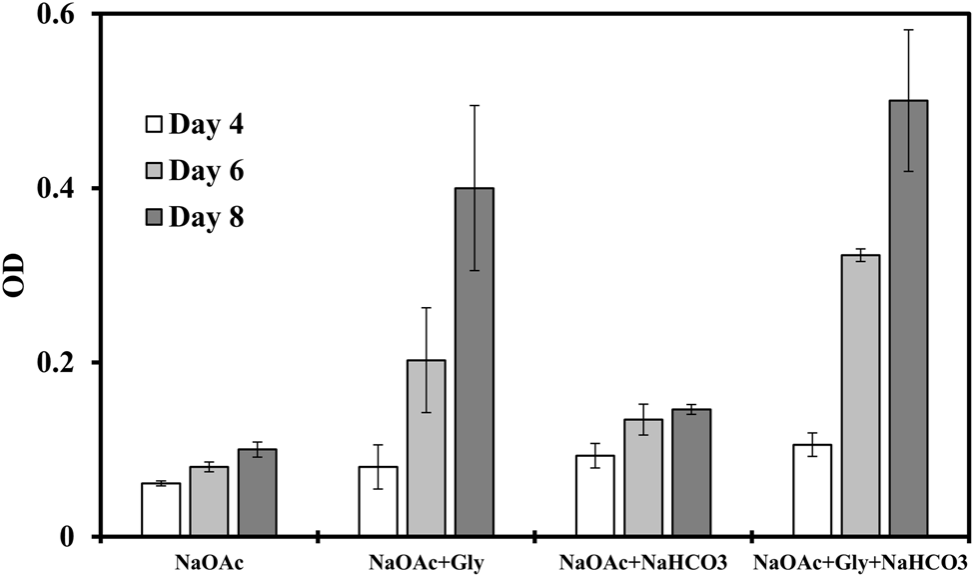
**Strain J-10-fl growth on sodium acetate (NaOAc, 2 g/L) in a minimal medium with or without glycine (0.05 g/L) addition under photoheterotrophic conditions.** NaHCO_3_ (0.5 g/L) was also supplemented to investigate its influence on the bacterial growth. Error bars represent the standard deviations of two biological replicates.

We then investigated the influences of these highly imported amino acids on the photoheterotrophic growth by replacing yeast extract with different amino acids. We observed the most significant growth enhancement (threefold to fivefold) when glycine was supplied (**Figure 2**), and a glycine concentration between 0.05 g/L and 0.25 g/L seems to suffice for enhancing the bacterial growth (Supplementary Figure S1). The influence of inorganic carbon was also investigated, which confirmed that addition of NaHCO_3_ (0.5 g/L) in medium slightly promoted photoheterotrophic growth, but much less than glycine did. Therefore, inorganic carbon source was not the rate limiting nutrients to the strain J-10-fl, and glycine played an important role in supporting the bacterial growth. These observations thus intrigued us to further investigate the role of glycine in *C. aurantiacus* metabolism.

**FIGURE 3:**
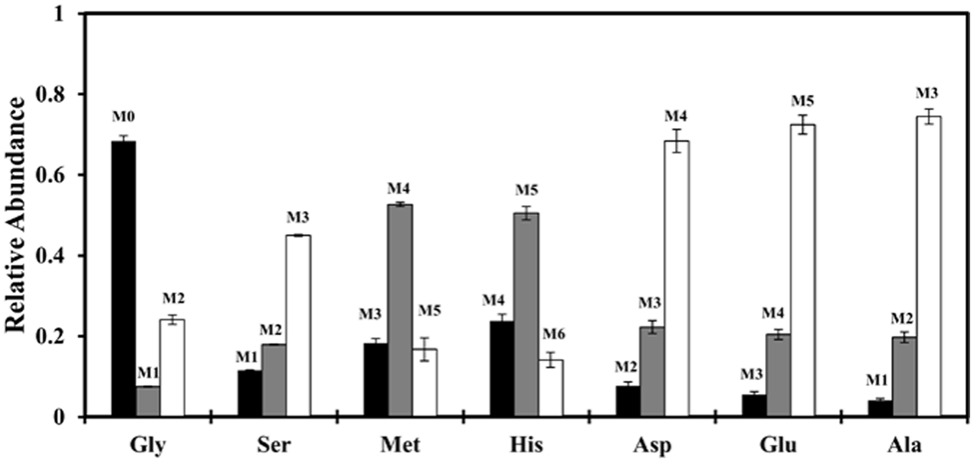
**Mass isotopomer distributions (MIDs) of selected proteinogenic amino acids ([M-57]^+^) under photoheterotrophic condition.** Strain J-10-fl was grown in a minimal medium supplied with [1,2-^13^C_2_] acetate (2 g/L) and unlabeled glycine (0.05 g/L). Error bars represent the standard deviations of two biological replicates.

To this end, we first traced the fate of glycine in the central carbon metabolism via ^13^C labeling experiments. We cultivated strain J-10-fl photoheterotrophically with [1,2-^13^C_2_]acetate and unlabeled glycine, and then measured the isotopomer distributions of proteinogenic amino acids. **Figure 3** shows that ^13^C from labeled acetate was incorporated into many key proteinogenic amino acids (e.g., Ala, Asp, and Glu) at significant levels (>90%), suggesting that glycine was not used as a carbon source for biomass synthesis. The only significant contributions of glycine to proteinogenic amino acids were glycine at 70% and serine (converted via glycine hydroxymethyltransferase, Caur_2543) at 30% (Supplementary Table S1). Substituting glycine with serine in minimal medium led to only a slight increase in cell growth (Supplementary Figure S1). Therefore, the profound growth enhancement by addition of glycine appears to be predominately due to factors other than direct conversion to biomass.

Glycine metabolism of *C. aurantiacus* has been previously investigated during photoautotrophic growth (Herter et al., 2001). The researchers have shown that: (1) *C. aurantiacus* does not generate glycine from glyoxylate (a product of the 3HOP pathway), and (2) it exhibits high enzyme activity to produce C1 units from glycine cleavage. C1 units, including 5, 10-methylene-tetrahydrofolate (5, 10-methylene-THF), 5-methyl-THF and 10-formyl-THF, participate in biosynthesis of amino acids and inosine monophosphate. For example, 5, 10-methylene-THF, derived from glycine degradation, can be converted to 5-methyl-THF and 10-formyl-THF. The former contributes to Met synthesis and the latter His synthesis. As for cultures growing with [1,2-^13^C_2_] acetate and unlabeled glycine, the most abundant isotopologs of the resulting proteinogenic Met and His have a single unlabeled carbon (**Figure 3**), which suggested that C1 units are mainly synthesized from glycine cleavage under photoheterotrophic conditions. Following is the glycine cleavage reaction (Kikuchi et al., 2008):

$$Glycine+TNF+{NAD}^{+}↔5,10-methylene-TNF+{CO}_{2}+{NH}_{3}+NADH+H^{+}$$

Glycine cleavage can also be coupled with the serine hydroxymethyltransferase reaction:

$$2Glycine+{NAD}^{+}+H_{2}O↔Serine+{CO}_{2}+{NH}_{3}+NADH+H^{+}$$

In fact, the glycine consumption rate was higher than its requirement as a carbon source for biomass growth (Supplementary Figure S2). Therefore, part of glycine must be cleaved and oxidized by the THF-dependent C1 pathway, which contains successive steps that oxidize 5, 10-methylene-THF to formate or CO_2_, generating both NADPH and ATP (Fan et al., 2014). The fate of glycine/C1-metabolism and the distribution of C1-metabolic enzymes/genes have been discussed for bacteria and archaea, including *Chloroflexi* (Braakman and Smith, 2012). These studies suggest that glycine cleavage and C1 degradation can serve as a key energetic route to produce ATP, NADH, and NADPH. Therefore, it is likely that glycine is actively involved in the energy metabolism of *C. aurantiacus* under photoheterotrophic conditions.

**FIGURE 4:**
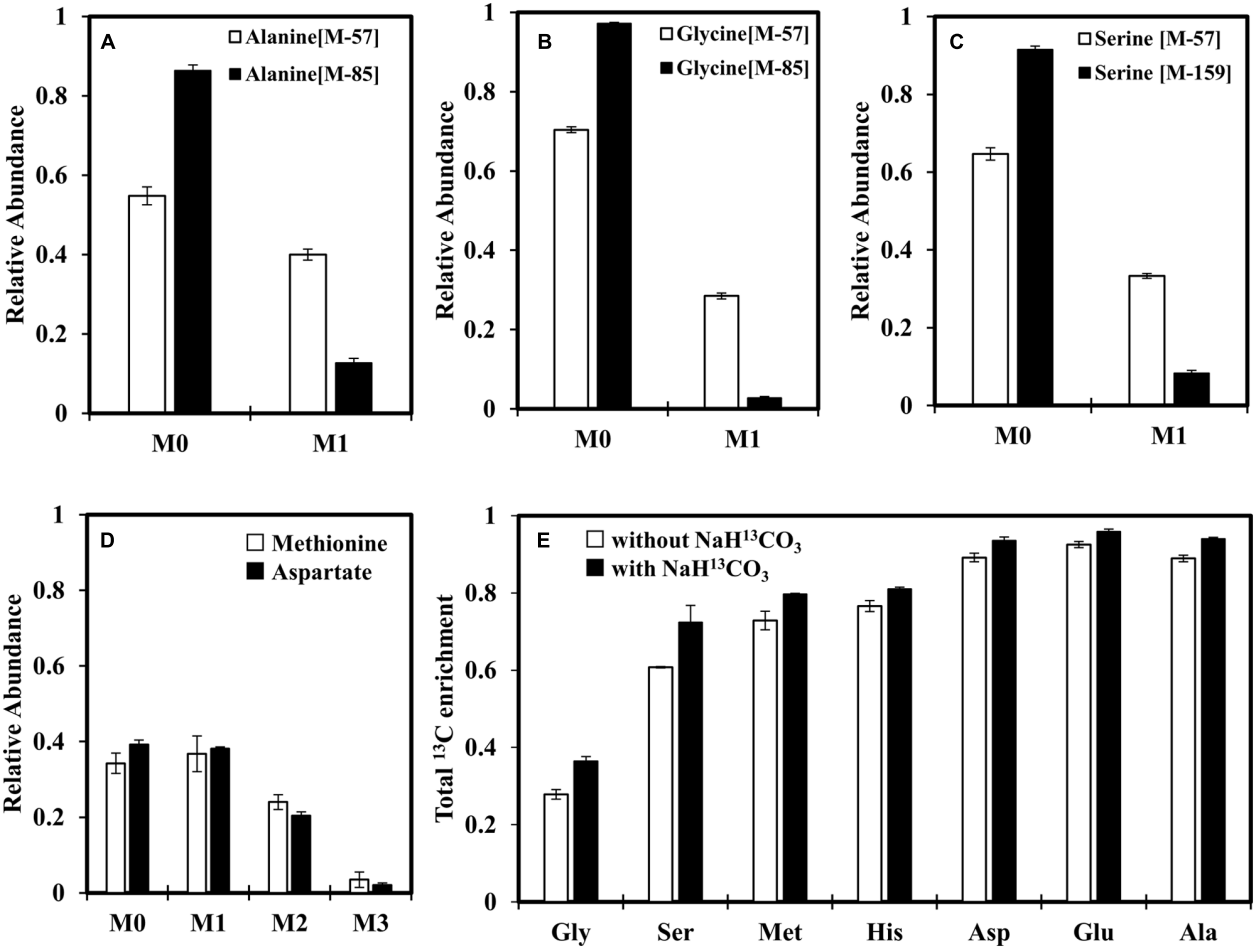
**Investigation of MID of proteinogenic amino acids to reveal CO_2_ fixation under photoheterotrophic conditions. (A–C)** MIDs of alanine, glycine and serine, respectively. **(D)** Comparison of MIDs between Met [M-57]^+^ and Asp [M-57]^+^. **(A–D)** Strain J-10-fl grew in a minimal medium supplied with unlabeled acetate (2 g/L), unlabeled glycine (0.05 g/L) and NaH^13^CO_3_ (0.5 g/L). **(E)** The total ^13^C enrichments of selected amino acids when J-10-fl was grown using [1,2-^13^C_2_] acetate (2 g/L) and unlabeled glycine (0.05 g/L) with or without NaH^13^CO_3_ (0.5 g/L) addition. Error bars represent the standard deviations of two biological replicates.

**FIGURE 5:**
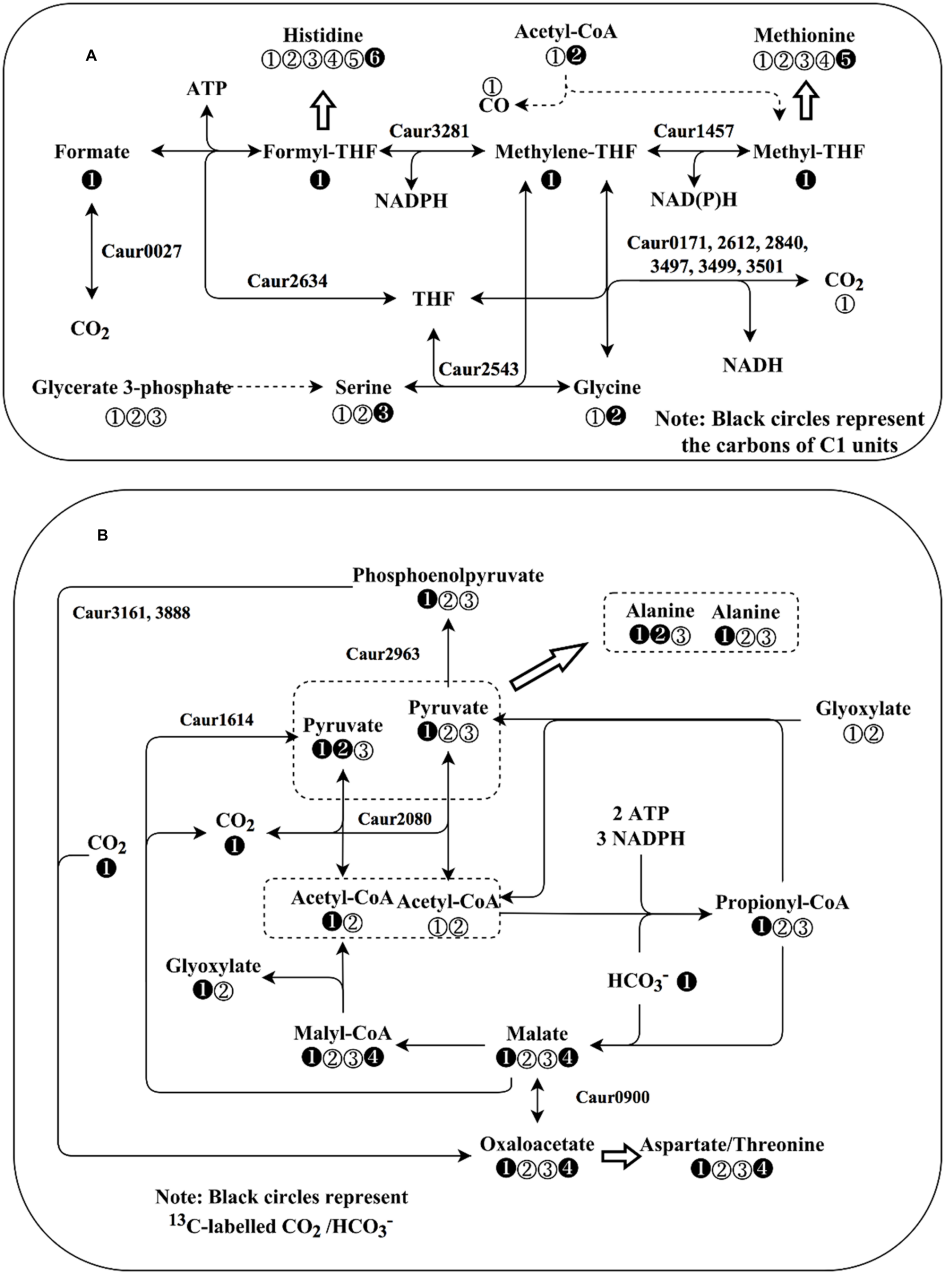
**Schematic representation of C1 metabolism and CO_2_ fixation pathways in *Chloroflexus aurantiacus*. (A)** C1 metabolism of *C. aurantiacus*. Black circles in **(A)** represent carbon atoms that will be donated to or originate from C1 unit carbon atoms. Dashed lines represent the genes that are not annotated in strain J-10-fl. Note: *C. aurantiacus* lacks the annotation of formate dehydrogenase gene, but it may use alternative enzyme (formylmethanofuran dehydrogenase, Caur_0027) for formate oxidation (Bertram et al., 1994). **(B)** Annotated CO_2_ fixation pathways in *C. aurantiacus*. The secondary CO_2_ fixation route (3HOP pathway) can generate acetyl-CoA of different labeling patterns. Black circles in **(B)** represent ^13^CO_2_ or H^13^CO_3_^-^. In both **(A,B)**, the numbers in the circles represent the positions of carbon atoms in corresponding intracellular metabolites.

In addition to glycine metabolism, we were also interested in investigating the CO_2_ fixation activity under photoheterotrophic conditions in the absence of H_2,_ as *C. aurantiacus* possesses multiple carbon fixation routes (Tang et al., 2011). To this end, we added NaH^13^CO_3_ (0.5 g/L) to minimal medium containing unlabeled acetate and glycine (**Figures 4A–D**). A protein BLAST search against either *Escherichia coli* or *Synechocystis* 6803 carbonic anhydrase on NCBI website suggests that the gene encoding carbonic anhydrase that converts H^13^CO_3_^-^ to ^13^CO_2_ is missing in *C. aurantiacus*, and an alternative ^13^CO_2_ source would be from the HCO_3_^-^-CO_2_ equilibrium in the culture medium (pH = 7.5). Although addition of NaHCO_3_ did not appear to promote strain J-10-fl growth (**Figure 2**), enzyme activities of CO_2_ fixation was measurable due to significant ^13^C incorporation into proteinogenic amino acids (e.g., ∼40% alanine is singly labeled and 5% alanine is doubly labeled, **Figure 4A** and Supplementary Table S1). As pyruvate is involved in the CO_2_ fixation pathways and also a precursor to alanine, the labeling patterns of alanine can reflect CO_2_ fixation routes (**Figure 5**). MS data show that alanine was mostly labeled at first position, and a small fraction of alanine was labeled at both first and second positions (Supplementary Table S1). **Figure 5B** shows the origins of different labeling patterns of alanine. Firstly, unlabeled acetyl-CoA and H^13^CO_3_^-^ can be condensed to [1-^13^C]pyruvate by pyruvate:ferredoxin oxidoreductase (PFOR, Caur_2080) or the 3HOP pathway. After a second H^13^CO_3_^-^ is incorporated in the 3HOP pathway, doubly labeled malyl-CoA is formed, subsequently resulting in the formation of [1-^13^C]acetyl-CoA via a cleavage reaction. When the PFOR reaction or the 3HOP pathway converts [1-^13^C] acetyl-CoA into pyruvate, [1,2-^13^C_2_] pyruvate is generated, which explains the origin of [1,2-^13^C_2_] alanine. Under anaerobic conditions without H_2_, the flux of the 3HOP pathway for CO_2_ fixation is weak, and [1,2-^13^C_2_] alanine only accounts for ∼5% of the total alanine (Supplementary Table S1), probably due to the lack of reducing equivalents to power this pathway. As a consequence, most [1-^13^C] pyruvate would be generated from the PFOR reaction, which also leads to production of [1-^13^C] serine and [1-^13^C] glycine (**Figures 4B,C**) after pyruvate is converted into downstream metabolites. **Figure 4D** shows similar labeling patterns between Met (derived from oxaloacetate and C1 unit) and Asp (derived from oxaloacetate), further confirming that most C1 units come from glycine degradation (**Figure 5A**). This phenomenon also indicates that ^13^CO_2_ is not preferred to be fixed via the reductive C1 pathway (CO_2_ ↔ formyl-THF ↔ 5, 10-methylene-THF) under photoheterotrophic conditions, as 5, 10-methylene-THF can be continuously generated from glycine. We conclude that reducing equivalents from glycine degradation are insufficient to drive appreciable CO_2_ fixation as part of promoting heterotrophic growth.

**Figure 4E** shows that ^13^C enrichment shifts of various amino acids after NaH^13^CO_3_ addition into minimal medium containing [1,2-^13^C_2_]acetate and unlabeled glycine. When strain J-10-fl was grown without NaH^13^CO_3_, 70% proteinogenic glycine and 40% proteinogenic serine were unlabeled. Other key proteinogenic amino acids (i.e., Ala, Asp and Glu) were labeled significantly (^13^C enrichments ∼90%). The small fraction of ^12^C in these amino acids was possibly from the metabolic assimilation of ^12^CO_2_ released from glycine degradation. When strain J-10-fl was cultivated with NaH^13^CO_3_, unlabeled glycine and fully labeled acetate, ^13^C enrichments in all proteinogenic amino acids were further raised (e.g., ∼95% of Glu carbons were ^13^C-carbons). This observation confirms photoheterotrophic CO_2_ co-utilizations by strain J-10-fl.

## Discussion

In this study, we attempted to understand why glycine can promote *C. aurantiacus* growth on acetate. Herter et al. (2001) have previously reported the contribution of glycine to photoautotrophic growth of *C. aurantiacus* strain OK-70-fl with H_2_/CO_2_. The study indicated that glycine could participate in serine synthesis and contribute approximately half of the C1 units to biomass synthesis. As a comparison, we tested the growth effect of glycine on strain OK-70-fl strain and revealed a similar growth enhancement (by threefold to fourfold) on strain J-10-fl during photoheterotrophic growth (Supplementary Figure S3 and related contents). Whether glycine can promote photoheterotrophic growth in the presence of H_2_ remains to be tested.

*Chloroflexus aurantiacus* does not contain a type I reaction center, and its cyclic photosynthetic electron transport system within the type-II reaction center generates ATP. We searched the genome database of strain J-10-fl and found the gene encoding a nickel-dependent hydrogenase (Caur_1188), which may generate NADPH in the presence of H_2_. However, when *C. aurantiacus* grows on acetate without H_2_, its NADPH generation could be less efficient. The major routes in central carbon metabolism for producing reducing power are the TCA cycle and oxidative pentose phosphate pathway (OPPP). However, acetate-based metabolism usually does not show strong fluxes through the OPPP, and both PFOR reaction and gluconeogenesis consume reducing equivalents. Additionally, genes encoding transhydrogenase have not yet been reported to exist in the genome of *C. aurantiacus.* Therefore, NADH and NADPH production from glycine cleavage and C1 degradation may increase energy flexibility and thus promote *C. aurantiacus* anaerobic growth. On the other hand, the THF-dependent C1 degradation pathway in *C. aurantiacus* appears to be influenced by H_2_. In our study, glycine cleavage contributes to C1 units for both Met and His synthesis under photoheterotrophic conditions. In comparisons, Herter et al. (2001) have investigated *C. aurantiacus* phototrophic growth with fully labeled glycine and H_2_/unlabeled CO_2_. Their labeling data of Met and His revealed that 5,10-methylene-THF (the C1 unit for Met synthesis) derived from fully labeled glycine was not converted to formyl-THF (the C1 unit for His synthesis). This phenomenon implied that H_2_ may inhibit the C1 unit oxidation, and that formyl-THF synthesis could be formed reductively from CO_2_ in the presence of H_2_. Lastly, it is possible that glycine may be also involved in other unknown mechanisms promoting *C. aurantiacus* photoheterotrophic growth. For example, a previous report has discovered the marine bacterium *Pelagibacter ubique* possesses all the genes for amino acid biosynthesis, but is still effectively auxotrophic for glycine (Tripp et al., 2009).

An interesting fact is that glycylglycine, a dipeptide of glycine, has been employed in the earliest studies of *C. aurantiacus* (Madigan et al., 1974), which gave the best growth of *C. aurantiacus* compared to other buffers (e.g., Tris, phosphate, and MOPS). Notably, glycylglycine is known to be a good buffer for biological systems since it is relatively non-toxic (Smith and Smith, 1949). In the absence of H_2_, *C. aurantiacus* grew much better in glycylglycine than in MOPS or other buffers (Supplementary Figure S3). Glycylglycine could be hydrolyzed at high temperature during medium autoclave and cell incubations (Radzicka and Wolfenden, 1996), or degraded by a membrane dipeptidase (Caur_2632). Our isotopic analysis further confirmed that *C. aurantiacus* growing on 100% [1-^13^C] acetate in the presence of unlabeled glycylglycine buffer possessed significantly unlabeled proteinogenic glycine (i.e., ∼90% of proteinogenic glycine was completely unlabeled, while only 3% of proteinogenic glutamate was completely unlabeled). Since the culture was grown in minimal medium containing ^13^C-acetate as the sole carbon source, the unlabeled glycine in the biomass must come from unlabeled glycylglycine. All these evidences imply that glycylglycine could be considered as an exogenous source of glycine contributing to growth enhancement for *C. aurantiacus*.

## Conclusion

In this study, we traced glycine in the central carbon metabolism to answer how it can enhance photoheterotrophic growth of *C. aurantiacus* in the absence of H_2_. Our results, together with previous studies and genome annotations, indicate that glycine can be used for producing biomass (mainly glycine and serine), but more importantly glycine molecules are degraded via cleavage reactions, serving as an important route for NAD(P)H production for acetate-grown *C. aurantiacus* cultures. As it is widely known, glycine is the simplest and also the most abundant amino acid that can be synthesized abiotically on the primitive Earth (Miller, 1953). Glycine cleavage and C1 metabolism might be the ancestral energy generation pathways (Braakman and Smith, 2012).

## Conflict of Interest Statement

The authors declare that the research was conducted in the absence of any commercial or financial relationships that could be construed as a potential conflict of interest.
