# Supplementary material for: Glycine Cleavage Powers Photoheterotrophic Growth of Chloroflexus aurantiacus in the Absence of H2
Source: Front Microbiol. 2015 Dec 22;6:1467. doi: 10.3389/fmicb.2015.01467 (PMC4686737; doi:10.3389/fmicb.2015.01467)
Supplement: Supplementary file 1 [file Data_Sheet_1.DOCX]

Glycine cleavage powers photoheterotrophic growth of *Chloroflexus aurantiacus* in the absence of H_2_

Lian He^1^, Yaya Wang ^2^, Le You^1^, Yadana Khin^2^, Joseph Kuo-Hsiang Tang ^2, 3,*^,Yinjie J. Tang^1,*^

^1^ Department of Energy, Environmental and Chemical Engineering, Washington University, St. Louis, MO 63130, USA.

^2^ Department of Chemistry and Biochemistry, Clark University, Worcester, MA 01610, USA

^3^ The Biodesign Institute, Arizona State University, Tempe, AZ 85281, USA

(Short title: glycine cleavage supports *Chloroflexus* growth)

*Corresponding authors:

Yinjie J. Tang: Email address: [yinjie.tang@seas.wustl.edu](mailto:yinjie.tang@seas.wustl.edu). Tel: 314-935-3441.

Joseph Kuo-Hsiang Tang: Email address: [Jktang@asu.edu](mailto:Jktang@asu.edu). Tel: 614-316-7886.

# Table S1 Mass isotopomer distributions of proteinogenic amino acids of *Chloroflexus aurantiacus* J-10-fl strain grown under photoheterotrophic conditions

| Carbon Sources | [1,2-^13^C] sodium acetate + glycine | | [1,2-^13^C] sodium acetate + glycine +NaH^13^CO_3_ | | sodium acetate + glycine +NaH^13^CO_3_ | |
| --- | --- | --- | --- | --- | --- | --- |
| Ion Fragments | Fractions | Standard Deviations | Fractions | Standard Deviations | Fractions | Standard Deviations |
| Alanine [M-57]' |  |  |  |  |  |  |
| 'M+0' | 0.02 | 0.00 | 0.01 | 0.00 | 0.55 | 0.02 |
| 'M+1' | 0.04 | 0.00 | 0.02 | 0.00 | 0.40 | 0.01 |
| 'M+2' | 0.20 | 0.01 | 0.10 | 0.01 | 0.05 | 0.01 |
| 'M+3' | 0.74 | 0.02 | 0.87 | 0.01 | 0.00 | 0.00 |
| 'Alanine [M-85]' |  |  |  |  |  |  |
| 'M+0' | 0.03 | 0.00 | 0.02 | 0.00 | 0.86 | 0.01 |
| 'M+1' | 0.11 | 0.01 | 0.07 | 0.00 | 0.13 | 0.01 |
| 'M+2' | 0.87 | 0.01 | 0.91 | 0.00 | 0.01 | 0.00 |
|  |  |  |  |  |  |  |
| 'Glycine [M-57]' |  |  |  |  |  |  |
| 'M+0' | 0.68 | 0.01 | 0.56 | 0.01 | 0.70 | 0.01 |
| 'M+1' | 0.07 | 0.00 | 0.14 | 0.00 | 0.29 | 0.01 |
| 'M+2' | 0.24 | 0.01 | 0.29 | 0.01 | 0.01 | 0.00 |
| 'Glycine [M-85]' |  |  |  |  |  |  |
| 'M+0' | 0.72 | 0.01 | 0.68 | 0.02 | 0.97 | 0.00 |
| 'M+1' | 0.28 | 0.01 | 0.32 | 0.02 | 0.03 | 0.00 |
|  |  |  |  |  |  |  |
| 'Serine [M-57]' |  |  |  |  |  |  |
| 'M+0' | 0.26 | 0.00 | 0.17 | 0.04 | 0.65 | 0.02 |
| 'M+1' | 0.12 | 0.00 | 0.09 | 0.01 | 0.33 | 0.01 |
| 'M+2' | 0.18 | 0.00 | 0.13 | 0.01 | 0.02 | 0.01 |
| 'M+3' | 0.45 | 0.00 | 0.60 | 0.04 | 0.00 | 0.00 |
| 'Serine [M-159]' |  |  |  |  |  |  |
| 'M+0' | 0.27 | 0.00 | 0.20 | 0.04 | 0.92 | 0.01 |
| 'M+1' | 0.21 | 0.00 | 0.16 | 0.01 | 0.08 | 0.01 |
| 'M+2' | 0.51 | 0.00 | 0.64 | 0.05 | 0.00 | 0.00 |
|  |  |  |  |  |  |  |
| 'Aspartate [M-57]' |  |  |  |  |  |  |
| 'M+0' | 0.01 | 0.00 | 0.01 | 0.00 | 0.39 | 0.01 |
| 'M+1' | 0.01 | 0.00 | 0.00 | 0.00 | 0.38 | 0.00 |
| 'M+2' | 0.08 | 0.01 | 0.04 | 0.01 | 0.20 | 0.01 |
| 'M+3' | 0.22 | 0.02 | 0.14 | 0.02 | 0.02 | 0.01 |
| 'M+4' | 0.68 | 0.03 | 0.81 | 0.03 | 0.00 | 0.00 |
| Aspartate f302' |  |  |  |  |  |  |
| 'M+0' | 0.04 | 0.00 | 0.03 | 0.00 | 0.59 | 0.01 |
| 'M+1' | 0.15 | 0.01 | 0.09 | 0.01 | 0.38 | 0.01 |
| 'M+2' | 0.81 | 0.01 | 0.89 | 0.01 | 0.02 | 0.00 |
|  |  |  |  |  |  |  |
| 'Glutamate [M-57]' |  |  |  |  |  |  |
| 'M+0' | 0.00 | 0.00 | 0.00 | 0.00 | 0.51 | 0.02 |
| 'M+1' | 0.00 | 0.00 | 0.00 | 0.00 | 0.41 | 0.01 |
| 'M+2' | 0.01 | 0.00 | 0.00 | 0.00 | 0.08 | 0.01 |
| 'M+3' | 0.06 | 0.01 | 0.03 | 0.01 | 0.01 | 0.00 |
| 'M+4' | 0.20 | 0.01 | 0.12 | 0.01 | 0.00 | 0.00 |
| 'M+5' | 0.72 | 0.02 | 0.84 | 0.02 | 0.00 | 0.00 |

Note: the standard deviations are based on biological replicates.

Table S1 Mass isotopomer distributions of proteinogenic amino acids of *Chloroflexus aurantiacus* grown under photoheterotrophic conditions (continued)

| Carbon Sources | [1,2-^13^C] sodium acetate + glycine | | [1,2-^13^C] sodium acetate + glycine +NaH^13^CO_3_ | | sodium acetate + glycine +NaH^13^CO_3_ | |
| --- | --- | --- | --- | --- | --- | --- |
| Ion Fragments | Fractions | Standard Deviations | Fractions | Standard Deviations | Fractions | Standard Deviations |
| 'Histidine [M-57]' |  |  |  |  |  |  |
| 'M+0' | 0.01 | 0.00 | 0.01 | 0.00 | 0.39 | 0.02 |
| 'M+1' | 0.01 | 0.00 | 0.00 | 0.00 | 0.41 | 0.00 |
| 'M+2' | 0.02 | 0.01 | 0.01 | 0.00 | 0.16 | 0.01 |
| 'M+3' | 0.07 | 0.01 | 0.04 | 0.00 | 0.04 | 0.01 |
| 'M+4' | 0.24 | 0.02 | 0.14 | 0.02 | 0.00 | 0.00 |
| 'M+5' | 0.51 | 0.02 | 0.62 | 0.03 | 0.00 | 0.00 |
| 'M+6' | 0.14 | 0.02 | 0.18 | 0.00 | 0.00 | 0.00 |
|  |  |  |  |  |  |  |
| 'Methionine [M-57]' |  |  |  |  |  |  |
| 'M+0' | 0.04 | 0.01 | 0.01 | 0.00 | 0.34 | 0.03 |
| 'M+1' | 0.02 | 0.01 | 0.01 | 0.00 | 0.37 | 0.05 |
| 'M+2' | 0.07 | 0.00 | 0.03 | 0.01 | 0.24 | 0.02 |
| 'M+3' | 0.18 | 0.01 | 0.11 | 0.00 | 0.03 | 0.02 |
| 'M+4' | 0.53 | 0.01 | 0.61 | 0.03 | 0.01 | 0.03 |
| 'M+5' | 0.17 | 0.03 | 0.23 | 0.02 | 0.01 | 0.01 |
|  |  |  |  |  |  |  |
| 'Valine [M-57]' |  |  |  |  |  |  |
| 'M+0' | 0.01 | 0.00 | 0.01 | 0.00 | 0.48 | 0.02 |
| 'M+1' | 0.01 | 0.00 | 0.00 | 0.00 | 0.41 | 0.01 |
| 'M+2' | 0.02 | 0.00 | 0.01 | 0.00 | 0.10 | 0.01 |
| 'M+3' | 0.07 | 0.01 | 0.04 | 0.00 | 0.01 | 0.00 |
| 'M+4' | 0.25 | 0.01 | 0.15 | 0.02 | 0.00 | 0.00 |
| 'M+5' | 0.64 | 0.02 | 0.79 | 0.02 | 0.00 | 0.00 |
|  |  |  |  |  |  |  |
| 'Leucine [M-15]' |  |  |  |  |  |  |
| 'M+0' | 0.01 | 0.00 | 0.01 | 0.00 | 0.60 | 0.02 |
| 'M+1' | 0.01 | 0.00 | 0.00 | 0.00 | 0.28 | 0.01 |
| 'M+2' | 0.01 | 0.00 | 0.01 | 0.00 | 0.09 | 0.01 |
| 'M+3' | 0.02 | 0.00 | 0.01 | 0.00 | 0.02 | 0.00 |
| 'M+4' | 0.09 | 0.02 | 0.05 | 0.01 | 0.00 | 0.00 |
| 'M+5' | 0.25 | 0.01 | 0.20 | 0.01 | 0.00 | 0.00 |
| 'M+6' | 0.60 | 0.05 | 0.72 | 0.02 | 0.00 | 0.00 |
|  |  |  |  |  |  |  |
| 'Isoleucine [M-15]' |  |  |  |  |  |  |
| 'M+0' | 0.01 | 0.00 | 0.01 | 0.00 | 0.32 | 0.01 |
| 'M+1' | 0.01 | 0.00 | 0.00 | 0.00 | 0.35 | 0.00 |
| 'M+2' | 0.02 | 0.00 | 0.01 | 0.00 | 0.23 | 0.01 |
| 'M+3' | 0.04 | 0.00 | 0.02 | 0.00 | 0.08 | 0.01 |
| 'M+4' | 0.12 | 0.02 | 0.07 | 0.01 | 0.02 | 0.01 |
| 'M+5' | 0.26 | 0.00 | 0.20 | 0.00 | 0.00 | 0.00 |
| 'M+6' | 0.55 | 0.03 | 0.69 | 0.00 | 0.00 | 0.00 |
|  |  |  |  |  |  |  |
| 'Phenylalanine [M-57]' |  |  |  |  |  |  |
| 'M+0' | 0.02 | 0.00 | 0.02 | 0.00 | 0.25 | 0.02 |
| 'M+1' | 0.01 | 0.00 | 0.00 | 0.00 | 0.35 | 0.01 |
| 'M+2' | 0.01 | 0.00 | 0.00 | 0.00 | 0.26 | 0.01 |
| 'M+3' | 0.01 | 0.00 | 0.00 | 0.00 | 0.11 | 0.01 |
| 'M+4' | 0.01 | 0.00 | 0.01 | 0.00 | 0.03 | 0.01 |
| 'M+5' | 0.03 | 0.01 | 0.01 | 0.00 | 0.01 | 0.00 |
| 'M+6' | 0.07 | 0.02 | 0.03 | 0.01 | 0.00 | 0.00 |
| 'M+7' | 0.16 | 0.02 | 0.09 | 0.01 | 0.00 | 0.00 |
| 'M+8' | 0.32 | 0.00 | 0.25 | 0.02 | 0.00 | 0.00 |
| 'M+9' | 0.37 | 0.05 | 0.59 | 0.04 | 0.00 | 0.00 |

# Estimation of fractions of proteinogenic alanine in different labelling patterns.

# In the labeling experiments, J-10-fl strain was grown under photoheterotrophic minimal medium containing unlabeled sodium acetate, glycine and NaH^13^CO_3_. The mass isotopomer distributions of different alanine ion fragments are shown below (also see Table S1):

| Ion Fragments | Fractions | Standard Deviations |
| --- | --- | --- |
| 'Alanine [M-57]^+^' |  |  |
| 'M+0' | 0.55 | 0.02 |
| 'M+1' | 0.40 | 0.01 |
| 'M+2' | 0.05 | 0.01 |
| 'M+3' | 0.00 | 0.00 |
| 'Alanine [M-85]^+^' |  |  |
| 'M+0' | 0.86 | 0.01 |
| 'M+1' | 0.13 | 0.01 |
| 'M+2' | 0.01 | 0.00 |
| ‘Alanine [f302]^+^' |  |  |
| 'M+0' | 0.55 | 0.02 |
| 'M+1' | 0.40 | 0.02 |
| 'M+2' | 0.05 | 0.00 |

The following table shows the eight isotopomers of alanine. Black circles represent ^13^C carbons, and white circles represent ^12^C carbons. The MID of ion fragments [M-57]^+^, [M-85]^+^ and [f302]^+^ can be used to calculate the fraction of alanine in each labelling pattern.

| Labeling patterns | variables | 'Alanine [M-57]^+^' | 'Alanine [M-85]^+^' | ‘Alanine [f302]^+^' |
| --- | --- | --- | --- | --- |
| ①②③ | X1 | 'M+0' | 'M+0' | 'M+0' |
| ❶②③ | X2 | 'M+1' | 'M+0' | 'M+1' |
| ①❷③ | X3 | 'M+1' | 'M+1' | 'M+1' |
| ①②❸ | X4 | 'M+1' | 'M+1' | 'M+0' |
| ❶❷③ | X5 | 'M+2' | 'M+1' | 'M+2' |
| ❶②❸ | X6 | 'M+2' | 'M+1' | 'M+1' |
| ①❷❸ | X7 | 'M+2' | 'M+2' | 'M+1' |
| ❶❷❸ | X8 | 'M+3' | 'M+2' | 'M+2' |

Thus, we can get the following equations:

X1=0.55;

X2+X3+X4=0.40;

X5+X6+X7=0.05;

X8=0.00;

X1+X2=0.86;

X3+X4+X5+X6=0.13;

X7+X8=0.01;

X1+X4=0.55;

X2+X3+X6+X7=0.40;

X5+X8=0.05;

The results are shown in the following table:

| Labeling patterns | variables | Fraction |
| --- | --- | --- |
| ①②③ | X1 | 0.55 |
| ❶②③ | X2 | 0.31 |
| ①❷③ | X3 | 0.09 |
| ①②❸ | X4 | 0.00 |
| ❶❷③ | X5 | 0.05 |
| ❶②❸ | X6 | 0.00 |
| ①❷❸ | X7 | 0.01 |
| ❶❷❸ | X8 | 0.00 |

Figure S1. Photoheterotrophic growth of J-10-fl strain in acetate-based medium with different concentrations of glycine (A) and serine supply (B).

Figure S2. Photoheterotrophic consumption of glycine by strain J-10-fl (with 0.05 g/L glycine supplement) (n=2, 10-day cultures).

Using GC/MS and TBDMS method (See Method and Materials), we measured glycine concentrations in the culture. A standard curve was first made by determining the relationship between the MS abundance and glycine concentration in medium (R^2^>0.99, Figure S2 A-B). We then used these curves to estimate the concentration of the remaining glycine in medium (Figure S2 C). A brief calculation shows that ~0.3 g/L of biomass would be produced within 10 days (Figure 2), which required ~7.5mg/L of glycine. However, ~40 mg/L has been absorbed from the medium, indicating much glycine is not used for biomass synthesis.

Figure S3. The growth of *C. aurantiacus* OK-70-fl in the acetate medium (without H_2_).

OK-70-fl grew under photoheterotrophic (light condition without O_2_) and chemoheterotrophic conditions (dark condition with O_2_). 20 mM acetate with or without 2 mM glycine was included in a minimal medium containing (Fig. A) glycylglycine or (Fig. B) MOPS buffer. Error bars represent the standard deviations of at least two biological replicates.


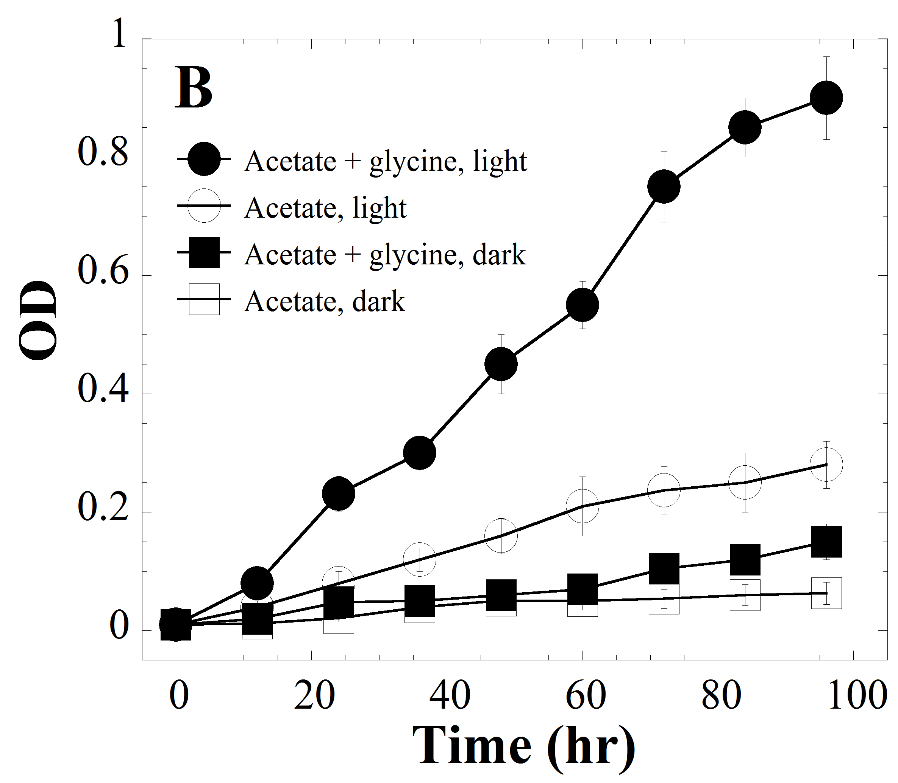

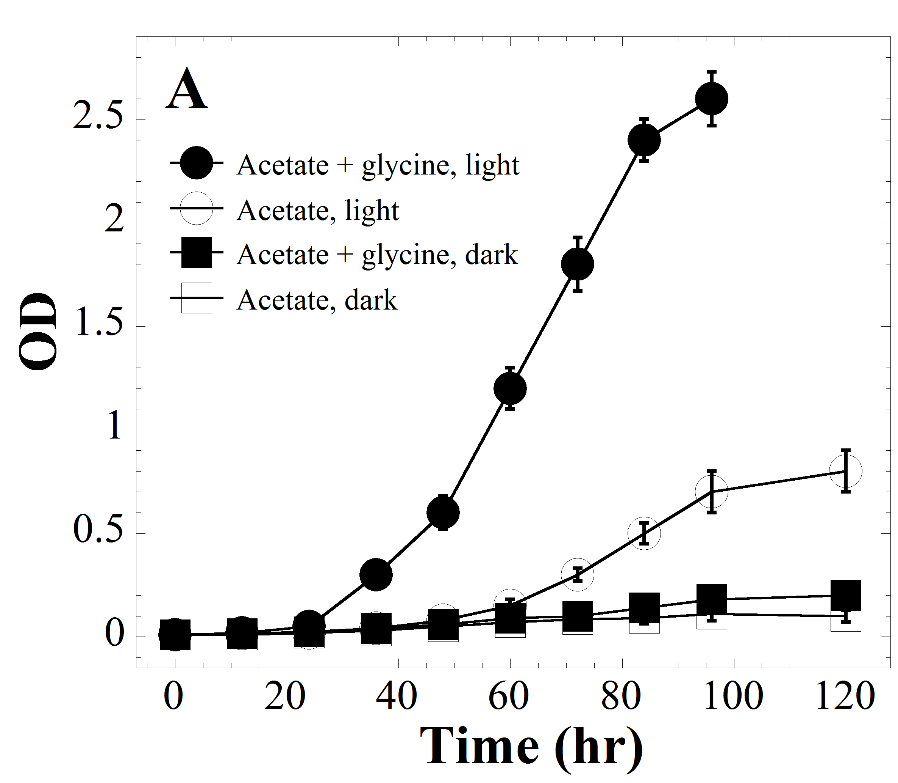


We also tested glycine impact on the growth of another *C. aurantiacus* strain (strain OK-70-fl) (48^o^C). Chemoheterotrophic OK-70-fl cultures were grown in shake flasks (180 rpm) under dark and aerobic conditions. Photoheterotrophic cultures were grown in the sealed bottles (without shaking) under continuous illumination at 20 *µ*mol photons m^-2^ s^-1^. Cells were cultured in “D” medium (One liter of medium containing 50 mL D stock), as described by (Hanada and Pierson 2006). One liter of D stock contains 10 mL Nitsch’s trace element solution, 10 mL FeCl_3_ solution (0.3 g L^-1^), 1.2 g CaSO_4_·2H_2_O, 2.0 g MgSO_4_·7H_2_O, 0.16 g NaCl, 2.06 g KNO_3_, 13.78 g NaNO_3_, 2.22 g Na_2_HPO_4_, 2.0 g nitrilotriacetic acid. One liter of Nitsch’s trace element solution contains 2.28 g MnSO_4_·H_2_O, 0.50 g ZnSO_4_·7H_2_O, 0.50 g H_3_BO_3_, 0.025 g CuSO_4_·5H_2_O, 0.025 g Na_2_MoO_4_·5H_2_O, 0.045 g CoCl_2_·6H_2_O. RPMI 1640 vitamins solution (100X, Sigma-Aldrich) was added into the sterile medium. In the culture medium, either glycylglycine (1 g/L) or MOPS (1g/L) was used as the buffer. To promote OK-70-fl growth, 0.05 g L^-1^ glycine was added into culture mediums.

Reference:

Hanada, S., and B. K. Pierson. 2006. The family chloroflexaceae. Prokaryotes 7:815-842.
